# Role of blood urea nitrogen in predicting the post-discharge prognosis in elderly patients with acute decompensated heart failure

**DOI:** 10.1038/s41598-018-31059-4

**Published:** 2018-09-10

**Authors:** Xiaohong Ren, Wei Qu, Lijuan Zhang, Miao Liu, Xuling Gao, Yuting Gao, Xiaodan Cheng, Weiwei Xu, Youhong Liu

**Affiliations:** Department of Cadre Health Care, Fourth People’s Hospital of Shenyang, Shenyang, 110031 China

## Abstract

Blood urea nitrogen (BUN) is a surrogate marker for neurohormonal activation, but the association between BUN and the post-discharge prognosis in elderly patients with acute decompensated heart failure (ADHF) is not well defined. We explored the association between BUN and post-discharge all-cause mortality in 652 elderly patients (73.9 ± 7.8 yr) with ADHF. All patients were followed for a mean duration of 32 months (12–69 months). BUN was analyzed both as a continuous variable and according to two categories: low BUN group (BUN < 15.35 mmol/L, N = 361) and high BUN group (BUN ≥ 15.35 mmol/L, N = 291). The risk of all-cause mortality increased by 1.6% per 1 mmol/L increase in BUN concentration when BUN was used as a continuous variable [hazard ratio (HR): 1.016, 95% confidence interval (CI): 1.006–1.026, *p* = 0.002]. BUN maintained an independent and significant positive correlation with all-cause mortality as a categorical variable (HR: 1.355, 95% CI: 1.023–1.794, *p* = 0.034 for the high BUN group). The BUN C-statistic for predicting all-cause mortality was 0.624 (95% CI: 0.585–0.661). The cut-off value for BUN was 15.35 mmol/L with sensitivity of 0.58 and specificity of 0.63. The prognostic performance of BUN was similar to brain natriuretic peptide (BNP) for predicting all-cause mortality (C-statistic: z = 0.044, *p* = 0.965). These results suggest that BUN is an independent predictor of post-discharge all-cause mortality in elderly patients with ADHF and its prognostic performance was similar to that of BNP.

## Introduction

Morbidity due to heart failure (HF) is increasing gradually^1^, and the incidence rate of newly occurring HF is far higher in the elderly population than in the young and middle-aged population^2^. Elderly patients with HF also have a worse prognosis than young and middle-aged patients^1,2^. Patients with acute decompensated heart failure (ADHF) usually suffer from high mortality after discharge^3,4^. Studies have shown that activation of the sympathetic nervous system (SNS), renin-angiotensin-aldosterone system (RAS), arginine vasopressin (AVP) and neurohumors are major pathophysiological changes in patients with HF^5,6^. The increased activities of the SNS and RAS enhance reabsorption of urea nitrogen by the proximal and distal renal tubules, and the increased secretion of AVP facilitates distribution of the urea transporter in the collecting ducts^5,6^. Therefore, blood urea nitrogen (BUN) is not only an indicator reflecting renal function, but also an effective marker indicating neurohormonal activation^5–7^. Previous studies have reported a significant correlation between an increase in BUN and a poor prognosis in patients with acute^8–14^ and chronic^15–17^ HF. However, no study has focused on the relationship between BUN and the post-discharge prognosis in elderly patients with ADHF. This study explored the effects of BUN on the post-discharge prognosis in elderly patients with ADHF.

## Results

### Baseline Characteristics of the Study Population by Clinical Outcome

The final study cohort consisted of 652 elderly patients with ADHF. The cohort was divided into a surviving group [418 patients (64.1%)] and a death group [234 patients (35.9%)]. The clinical characteristics of the two groups are shown in Table 1. The death group had significantly lower percentages of males, New York Heart Association (NYHA) class III, and discharge prescriptions of a β-receptor blocker and spironolactone, compared with the surviving group. Age, creatinine, uric acid, BUN, fasting blood glucose, brain natriuretic peptide (BNP), and heart rate were higher in the death group than those in the surviving group. Lower levels of red blood cells, hemoglobin, albumin, prealbumin, cholesterol, low density lipoprotein, serum sodium and ejection fraction were observed in the death group.

**Table 1:** Baseline Characteristics of the population by clinical outcome, median (IQR), or N (%), or means ± SD.

Variable	survival group (n = 418)	death group (n = 234)	Overall (n = 652)	*p*-value
Demographics				
Age, yrs	72.4 ± 7.3	76.5 ± 8.0	73.9 ± 7.8	<0.001
male	169 (40.4)	126 (53.8)	295 (45.2)	0.001
Medical history				
Ischemia cardiomyopathy	99 (23.7)	57 (24.7)	156 (23.9)	0.966
Diabetes Mellitus	98 (23.4)	60 (25.6)	158 (24.2)	0.530
Hypertension	250 (59.8)	134 (57.3)	384 (58.9)	0.527
Current smoking	113 (27.0)	64 (27.4)	177 (27.1)	0.930
Atrial fibrillation	59 (14.1)	32 (13.7)	91 (14.0)	0.877
Dilated cardiomyopathy	19 (4.5)	11 (4.7)	30 (4.6)	0.928
Valvular disease	29 (6.9)	14 (6.0)	0.222	0.637
Clinical Presentation				
NYHA class				<0.001
III	141 (33.7)	28 (12.0)	169 (25.9)	
IV	277 (66.3)	206 (88.0)	483 (74.1)	
SBP on admission, mm Hg	138.3 ± 25.3	138.9 ± 26.9	138.5 ± 25.9	0.783
DBP on admission, mm Hg	81.5 ± 14.5	80.2 ± 14.7	81.1 ± 14.6	0.262
Heart rate on admission, bpm	82.2 ± 22.3	88.2 ± 20.8	84.4 ± 21.9	0.001
Laboratory results on admission				
Leukocyte count (×10^9^/L)	7.09 ± 2.60	7.64 ± 3.13	7.29 ± 2.82	0.074
Hemoglobin, g/L	125.7 ± 20.2	119.2 ± 22.7	123.4 ± 21.3	0.001
Albumin, g/L	38.0 ± 4.1	35.9 ± 4.3	37.2 ± 4.3	<0.001
SGOT, U/L	18 (12, 29)	18 (11, 36)	18 (12, 32)	0.663
SGPT, U/L	22 (16, 37)	23 (15, 43)	22 (16, 38)	0.858
Creatinine, umol/L	81 (69, 100)	93 (74, 119)	84 (70, 106)	<0.001
Uric acid, umol/L	335 (257, 425)	370 (272, 488)	345 (259, 444)	0.001
BUN, mmol/L	13.92 (11.04, 17.82)	16.36 (12.72, 23.10)	14.64 (11.58, 19.46)	<0.001
Total cholesterol, mmol/L	4.45 ± 1.21	4.14 ± 1.18	4.34 ± 1.21	0.001
Low density lipoprotein, mmol/L	2.67 ± 0.93	2.40 ± 0.95	2.57 ± 0.94	0.001
High density lipoprotein, mmol/L	1.17 ± 0.37	1.14 ± 0.48	1.16 ± 0.41	0.304
Triglyceride, mmol/L	1.42 ± 1.00	1.30 ± 1.10	1.37 ± 1.03	0.177
fasting blood glucose, mmol/L	6.17 ± 1.84	6.56 ± 2.20	6.31 ± 1.98	0.005
serum potassium, mmol/L	4.07 ± 0.53	4.12 ± 0.63	4.09 ± 0.57	0.381
serum sodium, mmol/L	140.1 ± 3.9	138.0 ± 5.1	139.3 ± 4.5	<0.001
Troponin-I, ng/mL	0.04 (0.01, 0.47)	0.07 (0.02, 2.47)	0.05 (0.01, 0.83)	0.548
BNP, ng/L	752 (291, 1576)	1167 (607, 2345)	891 (363, 1759)	<0.001
Ejection fraction on admission%	52.3 ± 12.4	48.1 ± 13.2	50.8 ± 12.8	<0.001
Medical treatment at discharge				
ACEI/ARB	330 (78.9)	183 (78.4)	513 (78.7)	0.890
Beta-blockers	273 (65.3)	129 (55.1)	402 (61.7)	0.010
Spironolactone	276 (66.0)	129 (55.1)	405 (62.1)	0.006

NYHA, New York Heart Association; SBP, systolic blood pressure; DBP, diastolic blood pressure; bpm, beats per minute; SGOT, serum glutamic oxaloacetic transaminase; SGPT, serum glutamate-pyruvate transaminase; BUN, blood urea nitrogen; BNP, brain natriuretic peptide; ACEI/ARB, Angiotensin-converting enzyme inhibitors/Angiotensin receptor blockers.

### Prognostic Performance of BUN and BNP for the Prognosis Prediction

**Figure 1:**
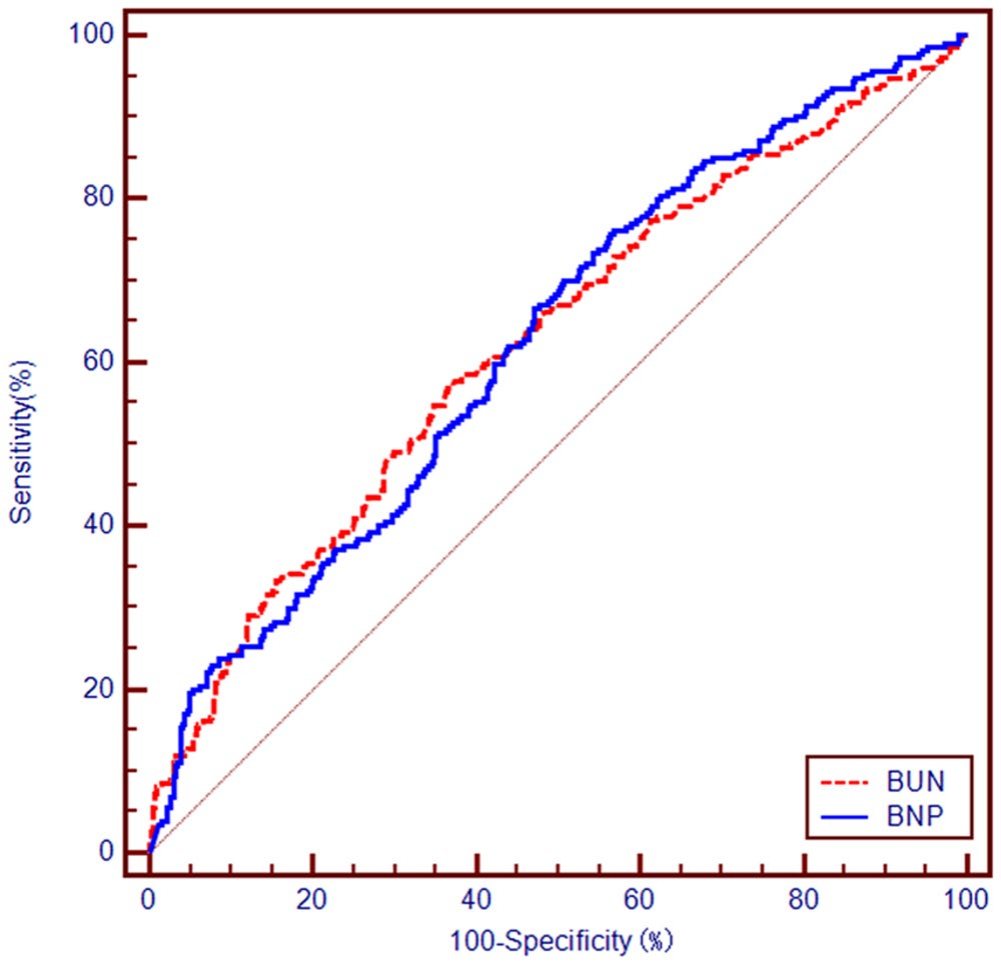
ROC curve analysis of BUN and BNP on the long-term prognosis of elderly patients with ADHF.

The C-statistics of BUN and BNP for predicting all-cause mortality were 0.624 [95% confidence interval (CI): 0.585–0.661] and 0.625 (95% CI: 0.587–0.662). The cut-off values for BUN and BNP for predicting all-cause mortality were 15.35 mmol/L with sensitivity of 0.58 and specificity of 0.63 and 805 ng/L with sensitivity of 0.67 and specificity of 0.53, respectively (Table 2, Fig. 1). The prognostic performance of BUN was similar to that of BNP (C-statistic: z = 0.044, *p* = 0.965) (Table 2).

**Table 2:** Prognostic performance of BUN and BNP for the prognosis prediction.

	C-statistic	Standard error	*p*-Value	95% CI	Difference	Z	*p*-Value
BUN	0.624	0.0231	<0.001	0.585–0.661	—	—	—
BNP	0.625	0.0226	<0.001	0.587–0.662	—	—	—
BUN vs. BNP	—	—	—	—	0.001	0.044	0.965

### Clinical Characteristics of the Study Population Based on BUN

According to the BUN cut-off value, 652 patients were divided into the low BUN group (BUN < 15.35 mmol/L, N = 361, 55.4%) and the high BUN group (BUN ≥ 15.35 mmol/L, N = 291, 44.6%). The clinical characteristics of the two groups are shown in Table 3. A higher proportion of males, NYHA class IV, and dilated cardiomyopathy were detected in the high BUN group but a lower proportion of hypertension and discharge prescriptions for β-receptor blockers and spironolactone was detected in the high BUN group than in the low BUN group. The high BUN group had lower diastolic and systolic blood pressure at admission and lower hemoglobin, albumin, total cholesterol, low density lipoprotein, high density lipoprotein, triglycerides, serum sodium, and ejection fraction, but had a higher age, creatinine, uric acid, potassium, and BNP than those in the low BUN group. The mortality rate in the high BUN group was significantly higher than that in the low BUN group during the follow-up (*46.4% vs 27.4%, p* < *0.001*).

**Table 3:** Clinical Characteristics of the population by BUN, median (IQR), or N (%), or means ± SD.

Variable	Low BUN group (n = 361)	High BUN group (n = 291)	*p*-value
Demographics			
Age, yrs	72.7 ± 7.7	75.3 ± 7.7	<0.001
male	137 (38.0)	158 (54.3)	<0.001
Medical history			
Ischemia cardiomyopathy	171 (47.4)	139 (47.8)	0.919
Diabetes Mellitus	85 (23.5)	73 (25.1)	0.648
Hypertension	229 (63.4)	155 (53.3)	0.009
Current smoking	99 (27.4)	78 (26.8)	0.860
Atrial fibrillation	47 (13.0)	44 (15.1)	0.442
Dilated cardiomyopathy	11 (3.0)	19 (6.5)	0.035
Valvular disease	24 (6.6)	19 (6.5)	0.951
Clinical Presentation			
NYHA class			<0.001
III	120 (33.2)	49 (16.9)	
IV	241 (66.8)	242 (83.1)	
SBP on admission, mm Hg	141.2 ± 25.5	135.2 ± 26.0	0.003
DBP on admission, mm Hg	82.5 ± 13.9	79.3 ± 15.3	0.006
Heart rate on admission, bpm	83.0 ± 21.4	86.0 ± 22.5	0.090
Laboratory results on admission			
Leukocyte count (×10^9^/L)	7.11 ± 2.59	7.51 ± 3.07	0.148
Hemoglobin, g/L	126.0 ± 18.7	120.0 ± 23.8	0.001
Albumin, g/L	38.1 ± 4.2	36.1 ± 4.1	<0.001
SGOT, U/L	18 (12, 30)	18 (12, 33)	0.790
SGPT, U/L	21 (16, 37)	24 (16, 43)	0.058
Creatinine, umol/L	76 (66, 90)	101 (82, 131)	<0.001
Uric acid, umol/L	308 (237, 398)	405 (295, 507)	0.001
Total cholesterol, mmol/L	4.54 ± 1.12	4.09 ± 1.26	<0.001
Low density lipoprotein, mmol/L	2.73 ± 0.96	2.37 ± 0.89	<0.001
High density lipoprotein, mmol/L	1.20 ± 0.41	1.10 ± 0.41	0.002
Triglyceride, mmol/L	1.48 ± 1.11	1.24 ± 0.91	0.003
fasting blood glucose, mmol/L	6.33 ± 1.95	6.28 ± 2.02	0.741
serum potassium, mmol/L	3.95 ± 0.45	4.26 ± 0.65	<0.001
serum sodium, mmol/L	140.2 ± 3.9	138.3 ± 4.9	<0.001
Troponin-I, ng/mL	0.04 (0.01, 0.71)	0.06 (0.02, 0.98)	0.897
BNP, ng/L	732 (290, 1455)	1168 (506, 2319)	<0.001
Ejection fraction on admission%	53.3 ± 12.1	47.7 ± 13.1	<0.001
Medical treatment at discharge			
ACEI/ARB	288 (79.8)	225 (77.3)	0.312
Beta-blockers	247 (68.4)	155 (53.3)	<0.001
Spironolactone	242 (67.0)	163 (56.0)	0.004
Clinical Outcome			
all-cause mortality,%	99 (27.4)	135 (46.4)	<0.001

NYHA, New York Heart Association; SBP, systolic blood pressure; DBP, diastolic blood pressure; bpm, beats per minute; SGOT, serum glutamic oxaloacetic transaminase; SGPT, serum glutamate-pyruvate transaminase; BUN, blood urea nitrogen; BNP, brain natriuretic peptide; ACEI/ARB, Angiotensin-converting enzyme inhibitors/Angiotensin receptor blockers.

### Prognostic Value of BUN for Determining Clinical Outcome

BUN was significantly predictive of all-cause mortality when used as a continuous variable [hazard ratio (HR): 1.029, 95% CI: 1.020–1.037, *p* < 0.001 for per 1 mmol/L increase] in the univariate Cox regression analysis (Table 4). After adjusting for covariates, BUN remained associated with all-cause mortality, with an increased all-cause mortality risk of 1.6% per 1 mmol/L increase in BUN concentration (HR: 1.016, 95% CI: 1.006–1.026, *p* = 0.002) (Table 4).

**Table 4:** Effects of multiple variables on Clinical Outcomes in COX regression analysis.

	Univariate Analysis			Multivariate Analysis		
	HR	95% CI	*p* value	HR	95% CI	*p* value
BNP per 1 ng/L increase	1.000	1.000–1.000	<0.001	1.000	1.000–1.000	0.001
Creatinine per 1 umol/L increase	1.000	0.997–1.003	0.997			
BUN as a continuous variable						
BUN per 1 mmol/L increase	1.029	1.020–1.037	<0.001	1.016	1.006–1.026	0.002^a^
BUN as a categories variable						
Low BUN group	Reference			Reference		
High BUN group	1.959	1.511–2.541	<0.001	1.355	1.023–1.794	0.034^a^

^a^Adjusted for age, gender, heart rate on admission, NYHA class, hemoglobin, albumin, uric acid, creatinine, total cholesterol, low density lipoprotein, fasting blood glucose, serum sodium, BNP, ejection fraction on admission, use of of β-receptor blockers and spironolactone.

When categorized into two groups (low BUN group: BUN < 15.35 mmol/L; high BUN group: BUN ≥ 15.35 mmol/L), BUN remained significantly predictive of all-cause mortality (Table 4). In the univariate Cox regression analysis, the high BUN group had a substantially higher risk of all-cause death compared with the low BUN group (HR: 1.959, 95% CI: 1.511–2.541, *p* < 0.001) (Table 4). In the multivariate Cox regression analysis, the high BUN group still conferred a significantly higher all-cause mortality than the low BUN group (HR: 1.355, 95% CI: 1.023–1.794, *p* = 0.034) (Table 4).

BNP independently predicted all-cause mortality in the univariate and multivariate Cox regression analyses (Table 4). However, creatinine was not an independent prognostic factor (Table 4).

**Figure 2:**
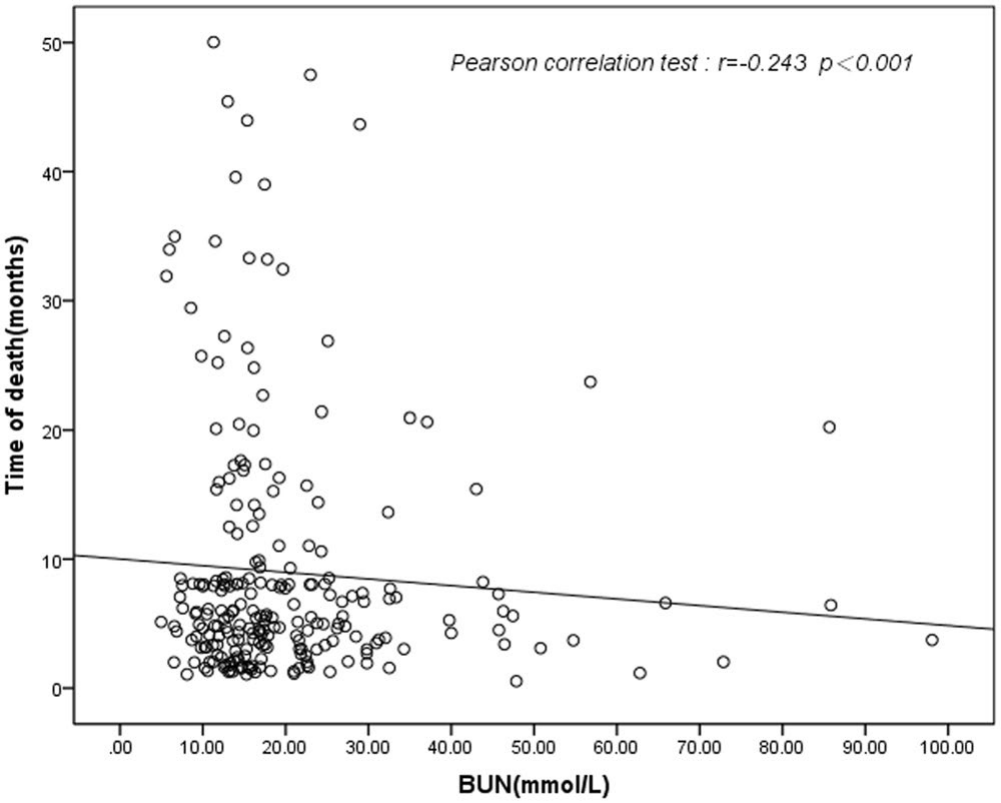
The correlation between BUN level and time of death.

Pearson’s correlation analysis revealed that time of death was significantly and negatively correlated with BUN level (r = −0.243, *p* < 0.001) (Fig. 2).

## Discussion

The present study tested the association between BUN and post-discharge all-cause mortality in elderly patients with ADHF. The main findings were as follows: (1) BUN was an independent predictor of post-discharge all-cause mortality in elderly patients with ADHF; (2) the prognostic performance of BUN was similar to that of BNP for predicting post-discharge all-cause mortality in elderly patients with ADHF.

BUN is a protein metabolic product of the human body that is synthesized in the liver and excreted by the kidneys. Thus, the BUN level represents the balance between urea production and renal excretion and is an important marker of renal damage. In the past, BUN has only been used to reflect renal function. For the first time, Aronson *et al*. studied the value of BUN for prognosing patients admitted for ADHF^8^. They found that BUN was an independent predictor of long-term all-cause mortality in patients admitted for ADHF^8^. This observation was verified and extended by other researchers who found that elevated BUN was an independent predictor of adverse outcomes in patients with acute^8–14^ and chronic^15–17^ HF. In addition, BUN was confirmed to be a stronger predictor of adverse outcomes than serum creatinine or estimated glomerular filtration rate (eGFR)^8,15,16^. However, no study has focused on the relationship between BUN and the post-discharge prognosis in elderly patients with ADHF. Our study confirmed that a high BUN was an independent predictor of all-cause mortality in elderly patients with ADHF. The risk of all-cause mortality increased by 1.6% per 1 mmol/L increase in BUN concentration when BUN was considered a continuous variable (HR: 1.016, 95% CI: 1.006–1.026, *p* = 0.002). BUN still maintained an independent and significant positive correlation with all-cause mortality as a categorical variable (HR: 1.355, 95% CI: 1.023–1.794, *p* = 0.034 for the high BUN group).

The pathophysiological association between BUN and adverse outcomes in patients with HF has been evaluated. First, HF activates the SNS and RAS, which can decrease eGFR and increase tubular urea reabsorption^5,6^. The RAS can increase the concentration-dependent reabsorption of urea by the proximal renal tubules, while the SNS can increase flow-dependent reabsorption of urea by the distal renal tubules. Previous studies have reported that BUN levels are correlated with the neurohumoral response^7,18,19^. Secondly, more AVP is secreted by patients suffering from HF, resulting in an increased distribution of the urea transporter in the collecting ducts, further causing an increase in urea reabsorption^5,6^. Thus, BUN may be a surrogate marker for activation of the SNS, RAS, and AVP.

BNP is a very important biomarker in patients with HF. It is widely recommended for the diagnosis, treatment, and the prognostic prediction of patients with HF^20,21^. In this study, both BUN and BNP independently predicted all-cause mortality. BUN was also proven to have the same discriminatory performance as BNP for predicting all-cause mortality (C-statistic: z = 0.044, *p* = 0.965). Taken together, BUN is a very useful clinical parameter to predict the long-term prognosis in elderly patients with ADHF, and can help us identify those patients at high risk for post-discharge all-cause death. These results emphasize the prognostic impact of BUN for post-discharge prognosis in elderly patients with ADHF.

This study had several limitations. First, this study was retrospective and observational, so potential confounders and selection bias could not be completely ruled out. Second, this study did not include all factors that influence BUN level, such as blood volume, nutritional state, gastrointestinal bleeding, and muscle wasting. Third, BUN was measured only at a single time-point (at admission), as studies have reported that patients with HF and a high BUN during hospitalization have a worse long-term prognosis^11,14,17^. Last, this study did not explore the effects of eGFR on the long-term prognosis in elderly patients with ADHF, because body weight data were lacking. However, this study confirmed that it was BUN, not eGFR or creatinine that independently predicted adverse outcomes in patients with ADHF^8^. Particularly, neurohormonal activation and hemodynamic abnormalities may play a prominent role in patients with ADHF^5,6^. BUN may increase because of activation of the SNS, RAS and AVP^5,6^, independently of changes in eGFR.

## Conclusions

BUN was an independent predictor of post-discharge all-cause mortality in elderly patients with ADHF. The prognostic performance of BUN was similar to that of BNP.

## Methods

### Study Design and Setting

This study was based on a retrospective cohort. In total, 670 consecutive elderly patients (age ≥ 60 years, average 73.9 ± 7.8 yr, 59.6% females), who were hospitalized for ADHF at a large hospital in Northeast China (Fourth People’s Hospital of Shenyang, Shenyang, China), were included in the cohort from January 2012 to January 2016. ADHF was defined according to guidelines^20,21^. All patients received standardized HF treatment according to the guidelines^20,21^. Patients who were receiving regular hemodialysis were excluded (n = 18). The final study cohort consisted of 652 patients. Clinical data of all cases were collected from the electronic medical records. Left ventricular ejection fraction was determined by echocardiography during hospitalization. In all cases, venous blood samples were drawn on admission into standard tubes and measured for BUN using a completely automated biochemistry-immunity analyzer (Ci 16200, Abbott, Abbott Park, IL, USA) in the core laboratory of the hospital. Clinical follow-up was assessed in January 2017 by a hospital visit or a phone interview of the patient’s general practitioner/cardiologist, the patient himself, or their family. All patients were followed for a mean duration of 32 months (12–69 months). The clinical endpoint of the study was all-cause mortality, which was identified from the patients’ medical records or the patient’s referring hospital physician. All events were validated by two independent event-judge physicians. This study complied with the Declaration of Helsinki, and the Fourth People’s Hospital of Shenyang Research Ethics Committee approved this research protocol. Written informed consent was formally obtained from all participants.

### Statistical Analysis

Quantitative variables are presented as mean ± standard deviation or median (interquartile range), and categorical variables are presented as counts and proportions (%). The Cox proportional-hazards regression model was used to analyze the effect of the variables on event-free survival. Variables showing significance in the univariate analysis (Table 1, *p* < 0.05) were entered into the final model, including age, gender, heart rate on admission, NYHA class, hemoglobin, albumin, uric acid, creatinine, total cholesterol, low density lipoprotein, fasting blood glucose, serum sodium, BNP, ejection fraction on admission, and use of β-receptor blockers and spironolactone. BUN was analyzed as a continuous and categorical variable. The results are reported as HRs with associated 95% CIs. The predictive performance of BUN and BNP was assessed by an index of discrimination (C-statistic). The C-statistic, which is defined by the area under the receiver operating characteristic curve in relation to all-cause mortality, was compared using a nonparametric test developed by DeLong *et al*.^22^ and MedCalc software for Windows, version 11.4.2.0 (MedCalc Software, Mariakerke, Belgium). Pearson’s correlation analysis was conducted to analyze the relationship between BUN level and time of death. All tests were two-sided, and a *p*-value < 0.05 was considered significant. All statistical analyses were performed with SPSS version 19 software (SPSS Inc., Chicago, IL, USA).

## Data Availability

All data generated or analyzed during this study are included in this published article.
